# Controlled Release of Doxorubicin for Targeted Chemo-Photothermal Therapy in Breast Cancer HS578T Cells Using Albumin Modified Hybrid Nanocarriers

**DOI:** 10.3390/ijms222011228

**Published:** 2021-10-18

**Authors:** Barbara Carrese, Chiara Cavallini, Gennaro Sanità, Paolo Armanetti, Brigida Silvestri, Gaetano Calì, Giulio Pota, Giuseppina Luciani, Luca Menichetti, Annalisa Lamberti

**Affiliations:** 1 Department of Molecular Medicine and Medical Biotechnology, University of Naples Federico II, 80131 Naples, Italy; barbara.carrese@unina.it; 2 Institute of Clinical Physiology, National Research Council, 56124 Pisa, Italy; chiara.cavallini@ifc.cnr.it (C.C.); paolo.armanetti@ifc.cnr.it (P.A.); 3 Institute of Applied Sciences and Intelligent Systems, National Research Council, 80078 Naples, Italy; gennaro.sanita@isasi.cnr.it; 4 Department of Chemical, Materials and Production Engineering, University of Naples Federico II, 80125 Naples, Italy; brigida.silvestri@unina.it (B.S.); giulio.pota@unina.it (G.P.); luciani@unina.it (G.L.); 5 Institute of Endocrinology and Experimental Oncology, National Research Council, 80131 Naples, Italy; g.cali@ieos.cnr.it

**Keywords:** doxorubicin, photothermal, breast cancer, PTT, hybrid nanoparticles, melanin nanoparticles, drug delivery

## Abstract

Hybrid nanomaterials have attracted research interest owing to their intriguing properties, which may offer new diagnostic options with triggering features, able to realize a new kind of tunable nanotherapeutics. Hybrid silica/melanin nanoparticles (NPs) containing silver seeds (Me-laSil_Ag-HSA NPs) disclosed relevant photoacoustic contrast for molecular imaging. In this study we explored therapeutic function in the same nanoplatform. For this purpose, MelaSil_Ag-HSA were loaded with doxorubicin (DOX) (MelaSil_Ag-HSA@DOX) and tested to assess the efficiency of drug delivery combined with concurrent photothermal treatment. The excellent photothermal properties allowed enhanced cytotoxic activity at significantly lower doses than neat chemotherapeutic treatment. The results revealed that MelaSil_Ag-HSA@DOX is a promising platform for an integrated photothermal (PT) chemotherapy approach, reducing the efficacy concentration of the DOX and, thus, potentially limiting the several adverse side effects of the drug in in vivo treatments.

## 1. Introduction

One of the most demanding challenges in cancer treatment is developing effective and safe strategies to achieve a targeted therapy [1,2,3,4,5]. In this regard, an encouraging strategy is represented by the development of nanoparticles (NPs) as carriers for drugs [1,2,3,4,5,6].

The use of NPs in targeted therapies allows overcoming different issues related to cancer treatment, including systemic side effects, off-target toxicity, aggregation, drugs’ low half-life, off-target uptake, and multidrug resistance [7,8].

In this context, targeted doxorubicin-loaded NPs were used to increase drug chemotherapeutic efficacy while reducing side effects through site-specific drug delivery. Indeed, doxorubicin (DOX), a potent topoisomerase II inhibitor that stalls cell cycle reducing cancer cell growth, is still one of the most used drugs to test new therapeutic approaches in various cancers, including breast cancer (BC) [9,10], the most frequent and dangerous female malignancy [11,12].

Combining conventional therapies, such as chemotherapy, with site-specific hyperthermia is an emerging strategy to improve cancer treatment [13,14]. Besides the direct impact on cell death, mild local heating can sensitize tumor cells to chemotherapy and enhance drug efficacy [15,16]. Nanoparticle-assisted photothermal therapy (PTT) relies on the ability of some materials to generate heat as result of light absorption. Among several classes of compounds, melanins [17] have shown good photothermal properties and ease of functionalization, representing promising bioinspired materials for tuning multifunctional therapeutic agents.

Recently, we developed hybrid eumelanin–silver NPs (MelaSil_Ag) functionalized with human serum albumin (HSA) as a new kind of nanoprobe (MelaSil_Ag-HSA NPs) able to target breast cancer cells via HSA–SPARC interaction [18]. MelaSil_Ag-HSA NPs demonstrated promising photoacoustic properties for molecular imaging in BC using safe non-ionizing radiation. By exploiting the physicochemical properties of these hybrid structures [19], MelaSil_Ag NPs were herein used to design DOX carriers (MelaSil_Ag-HSA@DOX NPs) for BC treatment. MelaSil_Ag-HSA@DOX NPs were evaluated for their pH controlled DOX release and PTT effectiveness, based on the synergistic effect of melanin and silver seeds [17], to confirm the efficacy of a more advanced chemotherapeutic approach.

## 2. Results and Discussion

### 2.1. Doxorubicin and Human Serum Albumin Interaction

The analysis of the fluorescence variation is an important method to determine the binding constant and mechanism and the number of binding sites for ligand–protein interactions. The binding between doxorubicin (DOX) and human serum albumin (HSA) is due to a static quenching mechanism through hydrophobic interactions and it is confirmed by the fluorescence decrease of a tryptophan residue (Trp-214) present in the DOX binding site of the protein [20].

**Figure 1:**
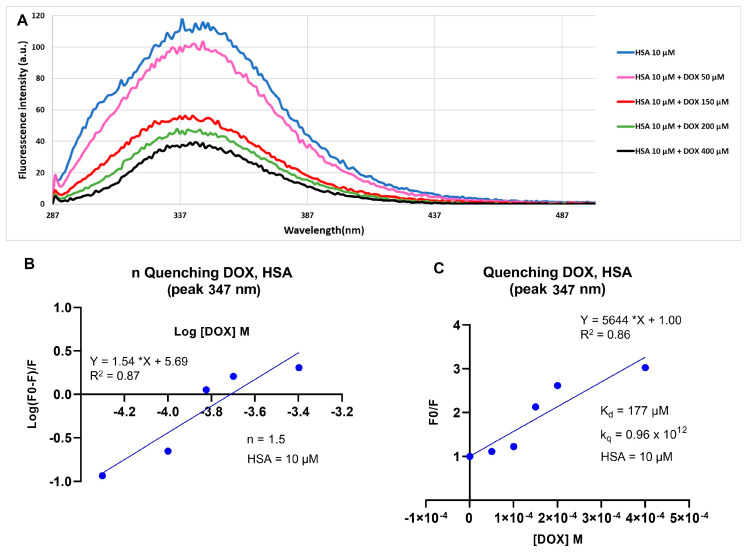
Fluorescence emission spectra and Stern–Volmer plots for HAS–doxorubicin interaction. (**A**) Fluorescence emission spectra; (**B**) Stern–Volmer plot of HSA binding sites; (**C**) Stern–Volmer plot of K_d_ and k_q_. *p* < 0.05.

To evaluate the DOX–HSA interaction, preliminary studies were performed. For this aim, HSA (10 µM) was analyzed without and with DOX at increasing concentrations up to 400 µM and the spectra were performed by using an excitation wavelength of 280 nm and collected from 287 to 500 nm (Figure 1A). Data showed that a good bonding capability was found at 400 µM of DOX, corresponding to a molar ratio HSA:DOX of 1:40. To calculate the number of binding sites (*n*), the quenching constant (k_q_) and the dissociation constant (K_d_), a Stern–Volmer plot of HSA fluorescence, quenched by increasing concentrations of DOX, and relative quenching parameters were evaluated. The *n* value, for each albumin molecule, was calculated by $log_{10}\left(\frac{F0-F}{F}\right)$ plotted versus $log_{10}$ of quencher molar concentration, which, in accordance with the literature, was 1.5 (Figure 1B) [20]. Furthermore, the quenching constant (k_q_ = 0.96 × 10^12^), which represents an index of the quenching efficiency, indicates a good efficiency of the quenching process (Figure 1C) and the value of K_d_ (177 µM) suggests a strong interaction between fluorophore and quencher.

### 2.2. DOX and MelaSil_Ag-HSA Nanoparticles Interaction

**Figure 2:**
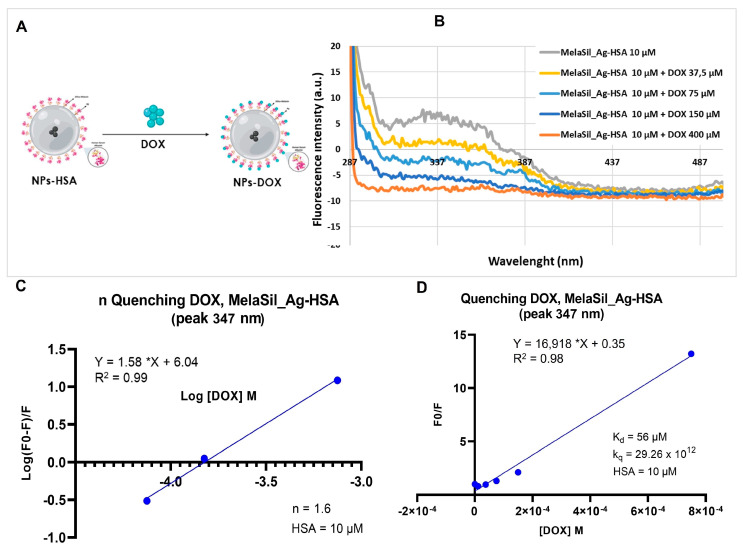
Fluorescence emission spectra and Stern–Volmer plots for MelaSil_Ag-HSA NPs—doxorubicin interaction. (**A**) DOX loading scheme; (**B**) fluorescence emission spectra; (**C**) Stern–Volmer plot of HSA binding sites; (**D**) Stern–Volmer plot of K_d_ and k_q_. *p* < 0.05.

To evaluate the interaction between DOX and HSA bonded to MelaSil_Ag NPs (Figure 2A), spectra in the absence and presence of DOX at increasing concentrations were performed (Figure 2B). The maximum bonding capability observed was at 400 µM of DOX and the kinetic parameters were calculated through Stern–Volmer relation (*n* = 1.6, k_q_ = 29.26 × 10^12^ and K_d_ = 56 µM) (Figure 2C,D). The higher value of k_q_ and the lower value of K_d_ compared to free HSA suggest the preservation of the tertiary structure of the protein bonded to the NPs’ surface and a better exposure of the DOX binding site.

### 2.3. DOX Loading and pH Dependent Release

The amount of DOX loaded to MelaSil_Ag-HSA NPs was calculated taking advantage of intrinsic fluorescence of DOX, as described in the Materials and Methods. The DOX bond efficiency and capacity was calculated for each preparation of MelaSil_Ag-HSA@DOX and, on average, the DOX concentration corresponding to 1 µg/mL of NPs was about 0.02 µM.

MelaSil_Ag-HSA NPs were characterized before and after loading of the drug by measuring the hydrodynamic diameter, polydispersity index (PDI), and ζ-potential. After DOX loading, a slight increase in the size of the particles from 394± 32 (PDI 0.26) to 407 ± 29 nm (PDI 0.45) was observed (Figure S1), with a ζ-potential of −27.2 ± 1.65 mV and −17 ± 2.16 mV, respectively (Table 1).

**Figure 3:**
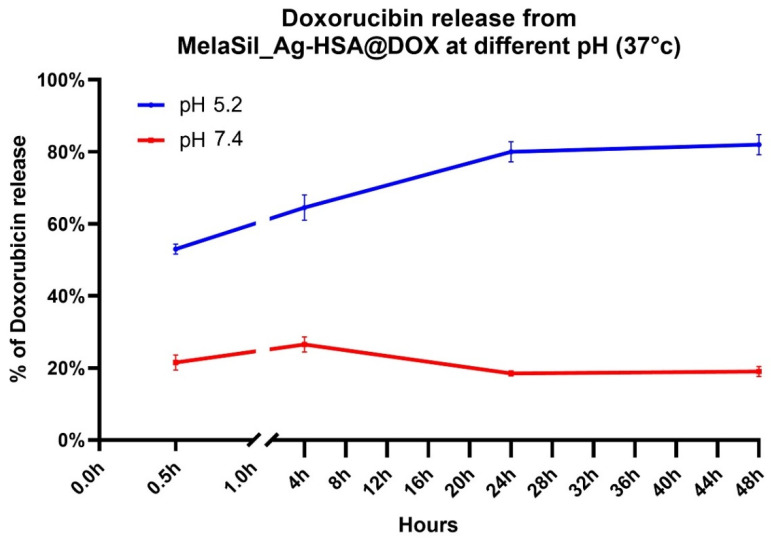
Doxorubicin release at pH 5.2 and pH 7.4. *p* < 0.05.

To evaluate DOX release from NPs-HSA@DOX in vitro, nanoparticles were incubated for increasing times at two pH (pH 5.2 and pH 7.4). At pH 5.2 in the first 30 min the release of DOX reaches 50% and up to 80% after 24 h. After that slower and sustained release has been observed. Otherwise, at pH 7.4 the maximum release of DOX (20%) was observed after 30 min to remain constant up to 48 h (Figure 3).

The different release behavior at two pH could be due to the acidic environment that increases DOX solubility, as well as induces a conformational change of HSA that reduces bonding capability [21]. Thus, the acidic pH of the tumor microenvironment could increase drug release by MelaSil_Ag-HSA@DOX and, thereby, reduce side effects to normal cells and organs [22].

### 2.4. DOX Delivery in HS578T Cells

**Figure 4:**
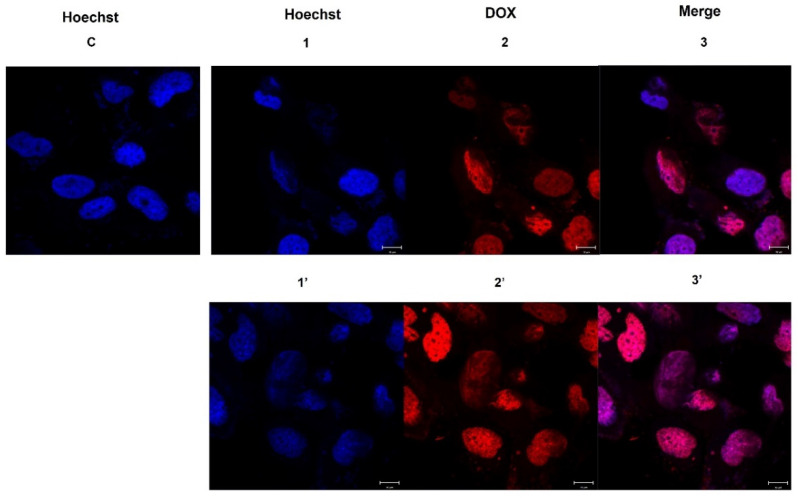
Representative images of confocal microscopy analysis of an HS578T cell line treated with NPs-HSA@DOX. (**C**) Control; (**1–3**) MelaSil_Ag-HSA@DOX 3 h; (**1′**–**3′**) MelaSil_Ag-HSA@DOX 6 h. Cell nuclei were stained with Hoechst 33258; DOX is visible as red color. Scale bar: 10 μm.

The uptake of MelaSil_Ag-HSA NPs loaded with DOX by the HS578T cell line was investigated by confocal microscopy. To this aim, cells were incubated with NPs-HSA@DOX with about 2.6 µM of the drug (NPs = 120 µg/mL) for 3 h and 6 h. The confocal microscopy images showed that there was a high degree of internalization in HS578T, according to the presence of DOX inside cell nuclei (Figure 4). The diffusion of DOX into nuclei was subsequent to targeted nanoparticle uptake and SPARC-mediated and pH-dependent drug release in the cytoplasm [18,21]. The combination of these two features could selectively increase the drug amount inside cancer cells by overcoming the multidrug resistance owing to a different internalization mechanism of the drug delivered by nanoparticles. Furthermore, it is well known that drug-loaded NPs have higher systemic circulation lifetime, better drug release kinetics, and selective tumor bioaccumulations with respect to the free drug having an important role in the overcoming of MDR [8].

### 2.5. Citotoxicity of MelaSil_Ag-HSA@DOX vs. Free DOX

**Figure 5:**
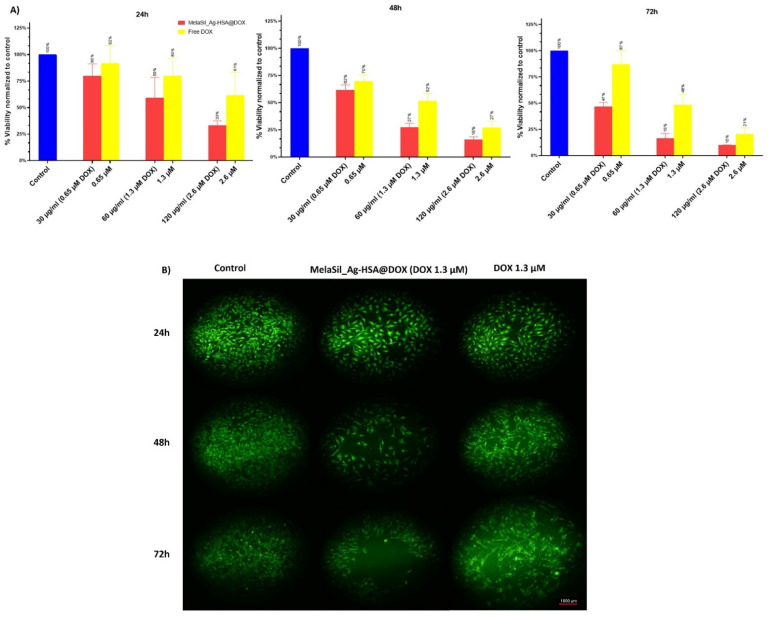
Cell viability assay. (**A**) CellTiter GLO assay of HS578T cells treated for 24 h, 48 h, and 72 h with MelaSil_Ag-HSA@DOX NPs (30, 60 and 120 μg/mL corresponded to DOX 0.65 µM, 1.3 µM, and 2.6 µM) and free DOX (at the same concentrations). *p* < 0.05; (**B**) Representative images of Calcein-AM fluorescent morphology images of HS578T cells before and after treatment with MelaSil_Ag-HSA@DOX NPs and free DOX. Scale bar 1000 μm.

To evaluate MelaSil_Ag-HSA@DOX NPs’ cytotoxicity compared to free DOX, CellTiter-GLO and Live Cell Explorer were performed. For CellTiter-GLO, HS578T breast cancer cells were incubated in the presence of increasing concentrations of free DOX (0.65 µM, 1.3 µM, and 2.6 µM) and DOX-loaded NPs (30 μg/mL, 60 μg/mL, and 120 μg/mL) for 24 h, 48 h, and 72 h. Results indicate that, in all tested conditions, MelaSil_Ag-HSA@DOX NPs were more toxic than the free drug. In particular, at 24 h of incubation, a viability reduction of 40% and 67% was observed at a concentration of DOX delivered by NPs equal to 1.3 µM and 2.6 µM, respectively, vs. 20% and 39% obtained with the same concentrations of free DOX. At 48 h of incubation, the difference in toxicity was even more evident when DOX at 1.3 µM was delivered by NPs compared to the free drug at the same concentration (73% vs. 48%). At the longest incubation time, the lowest DOX concentration (0.65 µM) carried by NPs showed a very good toxic effect (53%) when compared to the free DOX (13%) (Figure 5A). For Live Cell Explorer, HS578T cells were incubated with free DOX (1.3 µM) and MelaSil_Ag-HSA@DOX NPs (60 μg/mL) for increasing times. According with CellTiter-GLO, DOX-loaded NPs showed higher toxicity compared to the free drug (Figure 5B).

Furthermore, to evaluate general toxicity in healthy cells, a mammary breast fibrocystic disease cell line (MCF10a) was used. The results are reported in Figure S2 and clearly demonstrate a significant lower toxicity compared to breast cancer cells. The low toxic effect observed in MCF10a could be due to the release of a small amount of DOX in the culture medium at pH = 7.4.

Together, these results indicate that the use of nanoparticles to deliver chemotherapeutics allows the use of lower drug concentrations and exposure time to obtain better effects. This achievement is probably due to a targeted delivery, as demonstrated in our previous work [18], and to an overcoming of multidrug resistance due to the use of NPs as drugs carriers [8].

### 2.6. Thermal Properties of MelaSil_Ag-HSA NPs

**Figure 6:**
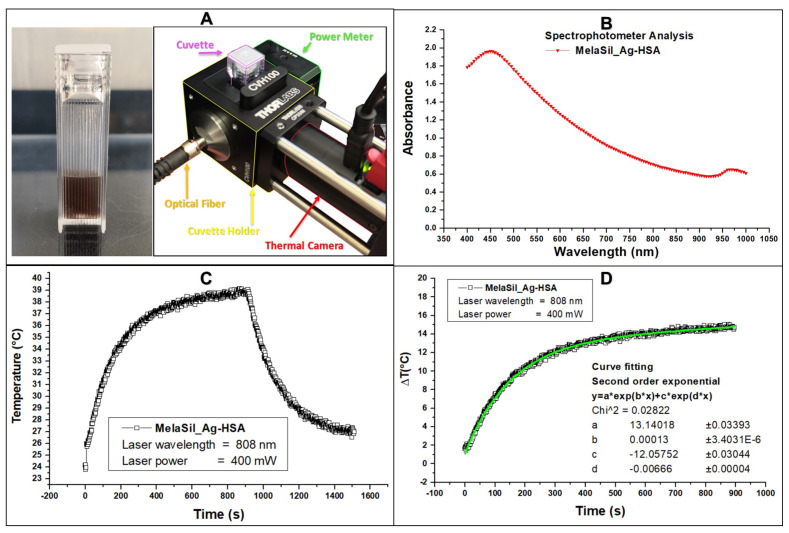
Thermal properties of MelaSil_Ag-HSA NPs. (**A**) MelaSil_Ag-HSA nanoparticles (1 μg/μL); (**B**) spectrophotometric analysis of the light absorbance properties of MelaSil_Ag-HSA; (**C**) typical thermal trends of MelaSil_Ag-HSA, CW heating (laser on), and cooling (laser off) processes; (**D**) dynamic evaluation of heating performances of MelaSil_Ag-HSA during laser illumination, and the fitting curve (green) of ΔT vs. time.

The light absorbance properties of the MelaSil_Ag-HSA (Figure 6A) were investigated by spectrophotometric analysis and reported in Figure 6B. MelaSil_Ag-HSA NPs showed an absorbance value around 0.69 at 808 nm wavelength. After 15 min of 808 nm laser illumination, MelaSil_Ag@HSA showed a temperature rise of 14 °C (Δ from 25 to 39 °C) (Figure 6C). The heating behavior was fitted according to a second-order exponential curve (f1):

∆T = a*e^b*t^ + c*e^d*t^ (f1) (1)

MelaSil_Ag-HSA NPs have been previously studied as bioinspired contrast agents for photoacoustic imaging [19], as well as targeting systems for BC cells [18]. Moreover, the optical absorbance given by the hybrid structure makes them suitable for therapeutic applications [23,24,25,26]. Indeed, under continuous wavelength (CW) laser stimulation, it is possible to achieve a ΔT (°C) temperature gradient up to 10 degrees centigrade in only 200 s. According to previous studies concerning different melanin nanoparticles [27], we wanted to classify the performances of MelaSil_Ag NPs in terms of photothermal properties. Indeed, because of the different hybrid compositions and structures, the physical effects and phenomena behaved differently, leading to improved thermal rise curves. In fact, during the laser stimulation, the MelaSil_Ag-HSA took advantage of the synergistic effect given by the combination of melanin and plasmonic metal seeds. The already known properties of melanin, such as light absorption, showed a dramatic increase in the presence of silver—we observed a thermal conversion efficiency of approximately up 30%, leading to an efficient thermal gradient at a melanin concentration of only 6 μg/mL in MelaSil_Ag-HSA NPs [19]. Compared to similar melanin-like nanoparticles, the performance of light absorption at 808 nm was improved, as demonstrated by the increase of the total photothermal efficiencies and the reduction of the efficacy concentration from 100 μg/mL of melanin, as reported in other studies with different nanostructures [23,25,27], to 6 μg/mL.

### 2.7. Citotoxicity of MelaSilAg-HSA@DOX upon Laser Irradiation

To investigate the effect of the combined NP administration and laser photothermal irradiation on HS578T breast cancer cell viability, cells were incubated at different times (3 h, 6 h, and 16 h) with increasing concentrations of MelaSil_Ag-HSA@DOX NPs or MelaSil_Ag-HSA and irradiated (3 W/cm^2^) for 5 min.

In this set of experiments, the combination of laser irradiation and free DOX was not explored based on preliminary experiments, indicating no significant absorbance of DOX at 808 nm (Figure S3), similarly to previous findings reporting comparable effects of free DOX with and without laser irradiation [27].

**Figure 7:**
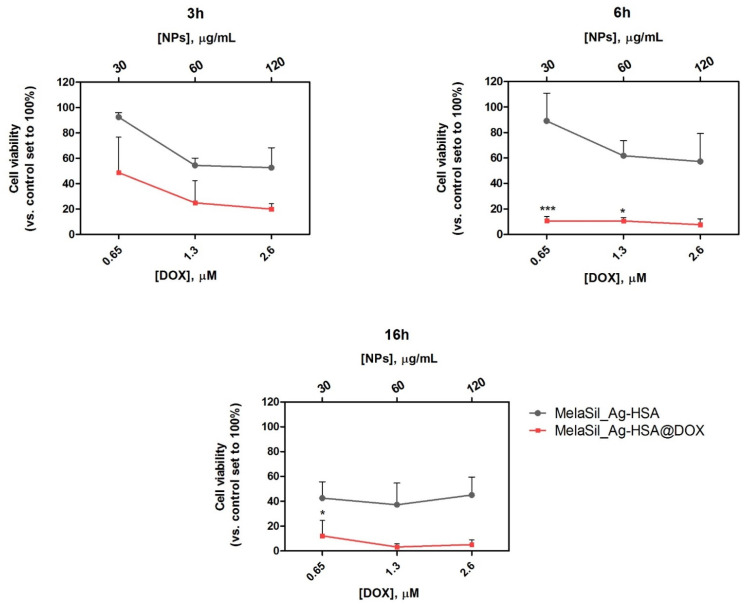
Cell viability of HS578T following 808 nm CW laser irradiation. Cells treated for 3 h, 6 h, and 16 h with MelaSil_Ag-HSA@DOX NPs (red) or MelaSil_Ag-HSA NPs (gray) at the concentrations of 30, 60, and 120 µg/mL (upper X axis), corresponding to 0.65, 1.3, and 2.6 µM DOX, respectively, for DOX-loaded NPs (lower X axis) and irradiated for 5′. * *p* < 0.05 and *** *p* < 0.001 versus cells treated with MelaSil_Ag-HSA. CellTiter-GLO assay was performed 24 h after irradiation.

The time points 6 h and 16 h were selected based on previous data [18] regarding the kinetic of MelaSil_Ag-HSA internalization in HS578T cells, while the shortest (3 h) was chosen based on several reports indicating that hyperthermia sensitizes cancer cells to chemotherapy, allowing a reduction in the exposure to nanoparticles [13]. Results given in Figure 7 indicate that the mutual chemo-photothermal treatment through MelaSil_Ag-HSA@DOX NPs was able to reduce cell viability at all the tested conditions. In particular, a dose-dependent decrease in cell viability was already observable for MelaSil_Ag-HSA@DOX NPs compared to MelaSil_Ag-HSA used at the same concentrations (51.3%, 75.0%, and 79.9% at 0.65 µM, 1.3 µM, and 2.6 µM DOX vs. 7.5%, 49.6%, and 58.4% at 30 μg/mL, 60 μg/mL, and 120 μg/mL, respectively) after a short incubation time with NPs (3 h). For longer incubation times (6 h and 16 h), a strong increase in cytotoxicity was assessed for all the tested concentrations. After 6 h of incubation, DOX-loaded NPs significantly reduced cell viability compared to HSA-NPs (80.2% vs. 8.4%, respectively, at 0.65 µM DOX). After 16 h of incubation, an increase of PTT efficacy was observed for MelaSil_Ag-HSA, as expected based on the internalization kinetics. Despite this, MelaSil_Ag-HSA@DOX NPs induced a higher cytotoxic effect compared to MelaSil_Ag-HSA (92.2% vs. 61.2%, respectively, at 0.65 µM DOX); indeed, the maximum relative cell damage was achieved at shorter incubation times compared to non-PTT treated cells.

The reduction of cell viability observed even in the presence of MelaSil_Ag-HSA confirms the ability of the NPs to act as photothermal agents and, thus, efficiently heat the cancer cells. The synergistic effect between the melanin and silver seeds enhanced the total photothermal effects, which was able to further promote PTT performances of this hybrid system, compared with conventional melanin nanomaterials. These results confirm that photothermally induced hyperthermia is a valuable strategy to improve the cytotoxic profile of MelaSil_Ag-HSA@DOX NPs.

## 3. Materials and Methods

### 3.1. Reagents

N-(3-dimethylaminopropyl)-N’-ethyl-carbodiimide hydrochloride (EDC), N-hydroxysuccinimide (NHS), 3-(aminopropyl)triethoxysilane (APTES), ethanol (absolute, ≥99.8%), doxorubicin HCl (DOX), and recombinant human serum albumin (rHSA) were purchased from Sigma-Aldrich (St. Louis, MO, USA). Phosphate-buffered saline (PBS) was purchased from Gibco (Grand Island, NY, USA). Live Cell ExplorerTM live-cell labeling kit was purchased from AAT Bioquest^®^ (Sunnyvale, CA, USA). CellTiter-GlO^®^ Luminescent Cell Viability Assay was purchased from Promega (Madison, WI, USA).

### 3.2. Cell Culture

Human breast carcinoma cell line (HS578T) and mammary breast fibrocystic disease cell line (MCF10a), obtained from the American Type Tissue Collection (Rockville, MD, USA), were grown in DMEM (GIBCO) and MEGM (Lonza, Basel, Switzerland), respectively. DMEM was supplemented with 10% heat inactivated FBS (GIBCO), 100 U/mL penicillin, 100 mg/mL streptomycin, and 1% L-glutamine. MEGM was supplemented with Mammary Epithelial Cell Growth Medium Bullet Kit (Lonza), 100 nM cholera toxin (Sigma-Aldrich) and 5% heat inactivated FHS (Lonza). All cell lines were grown at 37 °C in a 5% CO2 atmosphere.

### 3.3. HSA-DOX Quenching Effect

Human serum albumin (HSA) (10 µM) was incubated with DOX at increasing molar ratio HSA:DOX (1:5, 1:15, 1:20, and 1:40) in PBS 1× pH 7.4 for 24 h under stirring at room temperature (RT). The HSA fluorescence spectra were recorded with an excitation wavelength of 280 nm and recorded from 287 to 500 nm. All measurements were carried out in triplicate in three independent experiments.

### 3.4. Stern–Volmer Plots

To study the interaction between HSA and DOX and to calculate the dissociation constant (K_d_), the quenching constant (k_q_), and the number of binding sites (*n*), Stern–Volmer plot analysis was performed.

In brief, to calculate the K_d_ and k_q_ values, HSA fluorescence intensity at 347 nm (typical of tryptophan residues) was plotted as the ratio between fluorescence in the absence (F0) and in the presence (F) of the quencher (DOX) at various concentrations (50 μM, 150 μM, 200 μM, 400 μM) versus the molar concentrations of DOX. From the linear regression, the Stern–Volmer constant (KD) was determined as the slope of the straight of the modified Stern–Volmer equation $\frac{F0}{F}=1+k_{q}\tau \left[Q\right]=1+\mathrm{KD}\left[\mathrm{DOX}\right]$. The value of k_q_ was calculated as KD/τ (τ for static quenching of HSA = 5.9 ns) [20], k_d_ was calculated as 1/KD.

To determine the number of binding sites (*n*) of DOX to HSA, the log_10_(F0-FF) was plotted versus log_10_ of quencher molar concentration. After linear regression, the *n* value was determined as the slope of the straight from the equation $\log\frac{F0-F}{F}=\log1/k_{d}+n\log\left[DOX\right]$.

To assess the K_d_, K_q_, and *n* values of HSA–DOX interaction when albumin was bonded on MelaSil_Ag-HSA NPs, the same experimental setup used for free HSA was exploited, by using the MelaSil_Ag-HSA NPs corresponding to HSA 10 µM.

### 3.5. Evaluation of HSA Amount Bonded to MelaSil_Ag NPs

The amount of HSA bonded to NPs was determined by measuring the unbonded HSA after the functionalization process [18].

Unbonded HSA was precipitated through trichloroacetic acid (TCA) solution from every supernatant during the HSA functionalization protocol. The HSA precipitation was carried out by adding 10% *v*/*v* volume of TCA 100% to the supernatants. After 30′ at 4°C, the precipitate was collected by centrifugation at 12,000× *g* for 30′ at 4 °C, dried at 80 °C overnight (ON), and weighed with an analytical balance. The amount of HSA linked to MelaSil_Ag NPs was calculated as the subtraction of the unbonded albumin to the total amount used in the functionalization process. The percentage of HSA bonded to MelaSil_Ag NPs was obtained as (mg of bonded HSA/mg of MelaSil_Ag NPs) × 100.

### 3.6. Loading of DOX to MelaSil_Ag-HSA NPs

To load DOX on HSA modified NPs, MelaSil_Ag-HSA NPs (2.6 mg/mL) were added to DOX (with HSA:DOX ratio of 1:40) in PBS 1× for 96 h under stirring (24 h at RT and 72 h at 4 °C). Next, DOX-loaded NPs were collected by centrifugation and washed 3 times with PBS 1×. To calculate the DOX amount bonded to HSA NPs, a fluorescence calibration curve (Ex 480 nm, Em 590 nm) measured with a Multilabel Reader (PerkinElmer, Waltham, MA, USA) was used. The bonded DOX was obtained by subtracting the unbonded DOX from the total amount used. The DOX bond efficiency and capacity was calculated as (mg of bonded DOX/mg of total DOX) × 100 and (mg of bonded DOX/mg of MelaSilAg-HSA NPs) × 100, respectively.

### 3.7. Dynamic Light Scattering (DLS) and ζ-Potential Characterization

Size distribution and ζ-potential of MelaSil_Ag, MelaSil_Ag-has, and MelaSil_Ag-HSA@DOX were measured by a Zetasizer (Nanoseries, Malvern) using the laser dynamic scattering (λ = 632.8 nm) and the particle electrophoresis techniques, respectively. All the samples were diluted up to a droplet concentration of approximately 0.025% *w*/*v* by using Milli-Q water. A detecting angle of 173° and 5 runs for each measurement (1 run lasting 100 s) were used in the calculations of the particle size distribution. ζ-potential analyses were carried out by setting 50 runs for each measurement.

### 3.8. DOX Release from MelaSil_Ag-HSA@DOX

To evaluate the DOX release from loaded NPs at two different pH levels (5.2 and 7.4), MelaSil_Ag-HSA@DOX NPs were incubated at 2.6 mg/mL in PBS 1× at 37 °C under stirring for 0.5, 4, 24, and 48 h. Successively, the NPs were collected by centrifugation and the amount of released DOX was calculated by using a Multilabel Reader. The amount of DOX released by MelaSil_Ag-HSA@DOX was plotted as percentage of the total amount of DOX bonded to NPs.

### 3.9. Confocal Microscopy

HS578T cells (5 × 10^3^/coverslip) were plated on 10 mm glass coverslips positioned on the bottom of 24-well plate, allowed to attach for 24 h under normal cell culture conditions and then incubated with MelaSil_Ag-HSA@DOX NPs at 120 µg/mL (corresponding to 2.6 µM of DOX) for 3 and 6 h at 37 °C. Cells were washed with PBS, fixed in 4% formaldehyde for 20 min, and washed 3 times with PBS. Cell nuclei were then stained with Hoechst 33258 (Invitrogen, Carlsbad, CA, USA). Cells were then spotted on microscope slides and analyzed. Experiments were carried out on an inverted and motorized microscope (Axio Observer Z.1) equipped with a 63×/1.4 Plan Apochromat objective. The attached laser scanning unit (LSM 700 4× pigtailed laser 405–488–555–639; Zeiss, Jena, Germany) enabled confocal imaging. For excitation, 405 nm and 480 nm lasers were used. Fluorescence emission was revealed by Main Dichroic Beam Splitter and Variable Secondary Dichroic Beam Splitter. Double staining fluorescence images were acquired separately using ZEN 2012 software in the red and blue channels at a resolution of 512 × 512 pixels, with the confocal pinhole set to one Airy unit, and then saved in TIFF format.

### 3.10. Cell Viability of MelaSil_Ag-HSA@DOX versus Free DOX

For CellTiter-GLO assay, cells were seeded into 96-well microtiter plates (BD Falcon, United States) at the density of 5 × 10^3^ cells/well and incubated with free DOX at 0.65 µM, 1.3 µM, and 2.6 µM and MelaSil_Ag-HSA@DOX NPs were used at a concentration depending on the DOX loading efficiency and corresponding to the same DOX concentration (NPs ≅ 30, 60, and 120 µg/mL). The assay was performed after 24 h, 48 h, and 72 h of incubation for HS578T cells, according to the manufacturer’s instructions.

Luminescence was recorded for 0.25 s per well by a Multilabel Reader. For Live Cell Explorer, cells were seeded into 24-well microtiter plates at the density of 40 × 10^3^ cells/well and incubated with free DOX and MelaSil_Ag-HSA@DOX NPs in the same experimental conditions. The assay was performed according to the manufacturer’s instructions. After incubation, cells were observed by fluorescence microscopy and images were acquired.

For MCF10a cells, CellTiter-GLO assay was performed by using 1.3 µM and 2.6 µM of DOX carried by MelaSil_Ag-HSA@DOX NPs, for 24 h and 48 h.

### 3.11. Photothermal Response of MelaSil_Ag NPs under CW Laser Irradiation

The thermal behavior of MelaSil_Ag NPs was studied under prolonged laser illumination at 808 nm continuous wavelength (CW), a custom-made setup. In detail, 500 μL of NPs (1 μg/μL) were loaded in a polystyrene cuvette, then placed inside a cuvette holder, in which, on the left side was an optical fiber for laser illumination and on the right side was a power meter to measure the outgoing laser irradiation. The thermal infrared images were acquired during the NP heating (15 min) and cooling (10 min) steps, at a 10 Hz rate in matrices of 60 × 84 pixels, recording in time the absorbed power.

### 3.12. Cell Viability Following Laser Irradiation

For the PT experiments, cells were seeded into white 96-well plates at the density of 2 × 10^3^ cells/well. Then, cells were incubated with MelaSil_Ag-HSA@DOX or MelaSil_Ag-HSA NPs for 3 h, 6 h, or 16 h. MelaSil_Ag-HSA@DOX NPs were used at a concentration corresponding to 0.65 µM, 1.3 µM, and 2.6 µM DOX; MelaSil_Ag-HSA NPs were used at the same concentrations of MelaSil_Ag-HSA@DOX NPs. At the end of the incubation time, the medium was replaced with fresh medium and the cells were irradiated at 808 nm CW laser for 5 min, with a mean power density of 3 W/cm^2^. During irradiation, cells were maintained at 37 °C. Cell viability was assessed 24 h after irradiation by the CellTiter GLO assay, as described above.

### 3.13. Statistical Analysis

Results of the assays are expressed as mean ± SD of three independent experiments. Data are reported as average and SD. The statistical significance of differences among groups was evaluated using analysis of variance, using the software GraphPad Prism 9.0. The significance was accepted at the confidence level of 95% (*p* < 0.05).

## 4. Conclusions

We designed and tested, in a BC in vitro model, the system MelaSil_Ag-HSA NPs loaded with DOX and, subsequently, we explored their chemo- and photothermal efficiency. Our obtained results showed that DOX delivered by NPs is more effective than the free drug under the same experimental conditions, allowing the use of lower drug concentrations and time exposure with better effects. This might be due to the combined effect of targeted delivery and overcoming multidrug resistance. Furthermore, we investigated the effect of the DOX-loaded NP administration followed by photothermal laser irradiation. In this case, we observed a relatively higher cytotoxicity compared to dark conditions after 6 h of treatment at the lowest concentration. The enhanced cytotoxic efficacy could be due to a synergistic effect of temperature increase and doxorubicin toxicity, possibly increased by improved release owing to photothermal heating at 808 nm. These data confirm that photothermally induced hyperthermia is a viable strategy to improve the cytotoxic profile of MelaSil_Ag-HSA@DOX NPs. Moreover, the combined administration of chemo- and photothermal components represents a promising approach for reducing the timing of treatment thanks to their better efficacy. Overall, the results of this study pave the way for an in vivo application of these hybrid melanin-inspired nanomaterials, exploiting and tuning their structural and functional properties for cancer treatment.

## Figures and Tables

**Table 1:** DLS and ζ-potential of MelaSil_Ag-HSA NPs before and after DOX conjugation.

	ζ-Average (nm)	PDI	ζ-Potential (mV)
**MelaSil_Ag-HSA**	394 ± 32	0.26 ± 0.9	−27.2 ± 1.65
**MelaSil_Ag-HSA@DOX**	407 ± 29	0.45 ± 0.09	−17 ± 2.16

## Data Availability

All the data are included in the figures.

## Supplementary Materials

The following are available online at www.mdpi.com/article/10.3390/ijms222011228/s1.
