# Supplementary material for: Controlled Release of Doxorubicin for Targeted Chemo-Photothermal Therapy in Breast Cancer HS578T Cells Using Albumin Modified Hybrid Nanocarriers
Source: Int J Mol Sci. 2021 Oct 18;22(20):11228. doi: 10.3390/ijms222011228 (PMC8538307; doi:10.3390/ijms222011228)
Supplement: Supplementary file 1 [file ijms-22-11228-s001.zip › ijms-1423795-supplementary.pdf]

Supplementary Materials: Controlled Release of Doxorubicin for Targeted Chemo-Photothermal Therapy in a Breast Cancer HS578T Cells using Albumin Modified Hybrid Nanocarriers

Barbara Carrese [1,†], Chiara Cavallini [2,†], Gennaro Sanità [3], Paolo Armanetti [2], Brigida Silvestri [4], Gaetano Calì [5], Giulio Pota [4], Giuseppina Luciani [4], Luca Menichetti [2,*] and Annalisa Lamberti [1,*]

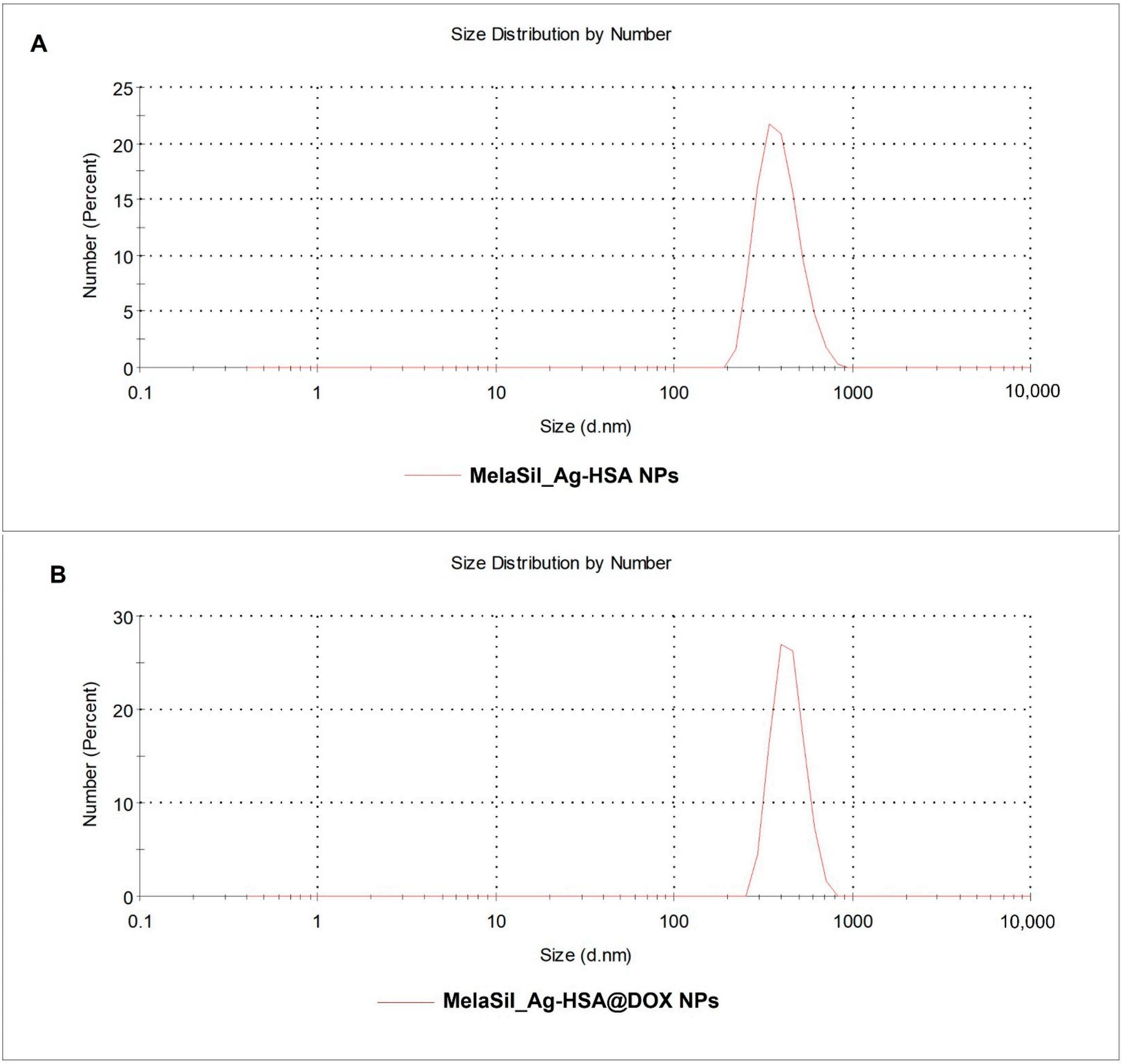

Figure S1. Number distribution (%) of DLS measurements on (**A**) MelaSil_Ag-HSA NPs at 10, 0 μg/mL and (**B**) MelaSil_Ag-HSA@DOX NPs at 100 μg/mL.

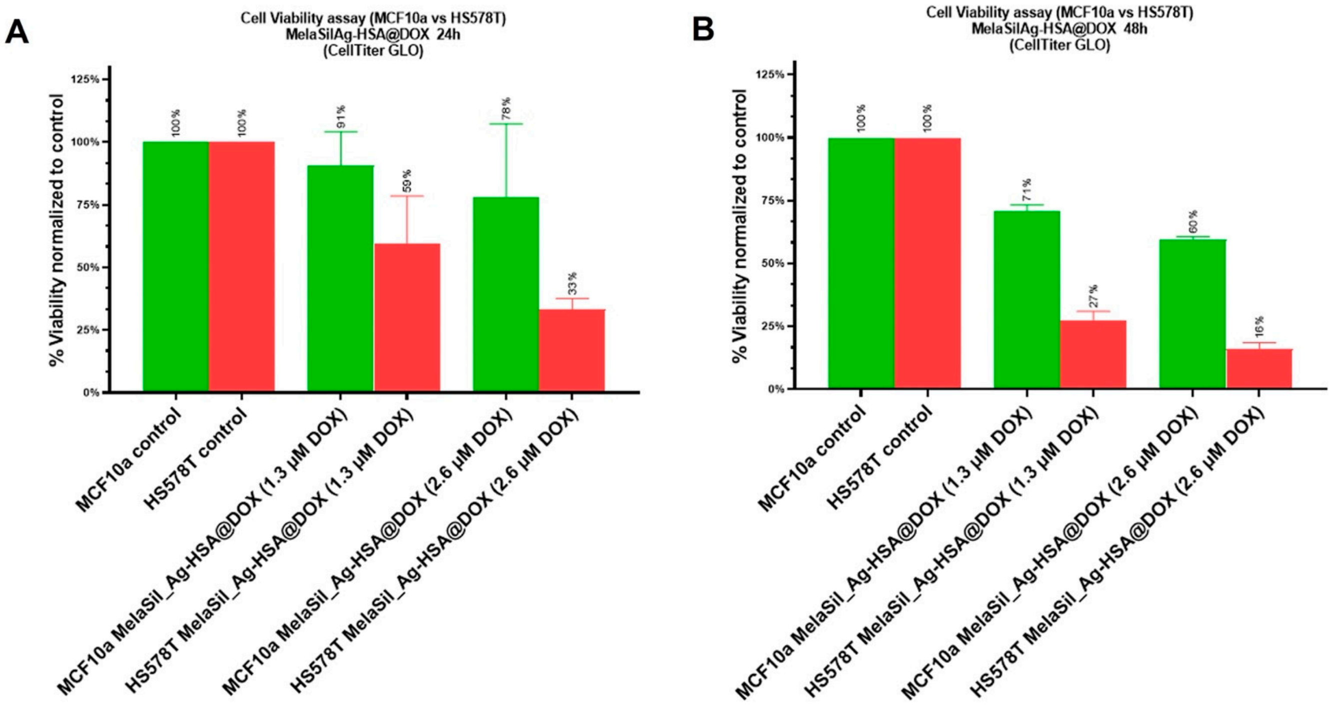

Figure S2. Cell viability assay. Cell-Titer GLO assay of HS578T and MCF10a cells treated for 24 h **(A)** and 48 h **(B)** with MelaSil_Ag-HSA@DOX NPs. P< 0.05

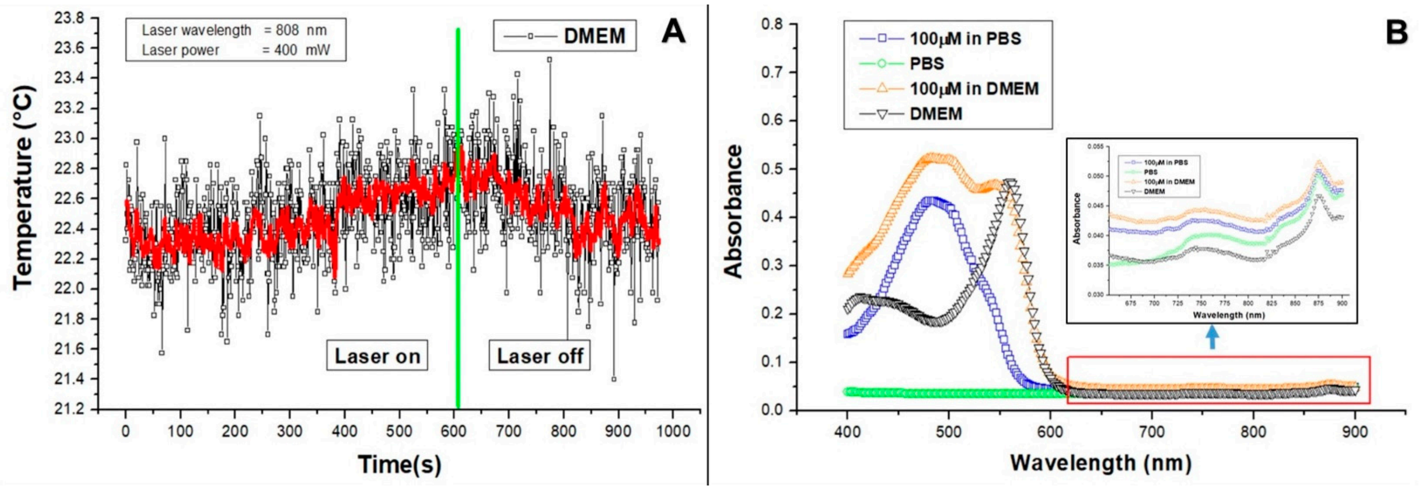

Figure S3. **(A)** Thermal trend of DMEM culture medium as a function of 808 nm laser irradiation (400 mW) time. CW heating (laser on) and cooling (laser off) processes. **(B)** Spectrophotometric analysis of the light absorbance properties of free DOX (100 μM) in DMEM (orange), free DOX (100 μM) in PBS (blue), DMEM (black), and PBS (green)
